# Differences in medical education before, during, and in the post-peak period of the COVID-19 pandemic—exploring senior medical students’ attitudes

**DOI:** 10.1186/s12909-023-04489-6

**Published:** 2023-07-13

**Authors:** Bryan F. Vaca-Cartagena, Erika Quishpe-Narváez, Heidi Cartagena Ulloa, Jenny Paola Estévez-Chávez

**Affiliations:** 1 Breast Cancer Center, Hospital Zambrano Hellion TecSalud, Tecnologico de Monterrey, San Pedro Garza Garcia, Nuevo Leon Mexico; 2 Pontificia Universidad Católica del Ecuador, 12 de Octubre #1076, Quito, Pichincha Ecuador

**Keywords:** Education, Medical, Students, Medical, Schools, Medical, COVID-19, SARS-CoV-2

## Abstract

**Background:**

The burden that COVID-19 has brought to the economy, healthcare systems, and education is unmatched. Public health and social measures were implemented to halt transmission. Thus, social gathering and in-person learning, core aspects of medical education, were interrupted. Studies have documented the detrimental impact students graduating during the pandemic have had on their confidence and skills. However, data comparing pre-pandemic, pandemic, and post-peak students still lack. This study aimed to identify senior medical students' attitudes regarding their education and compare them according to the three previously described periods.

**Methods:**

In this cross-sectional study, the survey employed was designed based on a previous questionnaire and applied to senior medical students before graduating between January 2018 and June 2022. Answers were collected using a three-point Likert scale and Yes/No questions. Associations between variables were examined using Chi-squared, Fisher’s Exact tests, and ANOVA, employing logistic regression to calculate odds ratio (OR) when appropriate.

**Results:**

A total of 679 responses were analyzed. Most students (59%) were women. Up to 383, 241, and 55 senior medical students answered the survey before, during, and in the post-peak period of the COVID-19 pandemic, respectively. There was a staggering decrease in the percentage of students in the post-peak compared to the pre-pandemic period that considered certain factors such as being taught about the doctor-patient relationship (62% vs 75%), practicing teamwork (33% vs 54%), preclinical & clinical subjects (44% vs 63%), and being taught to conduct research (22% vs 32%) as “very useful” to their professional traineeship. There was a significant difference between pre-pandemic, pandemic, and post-peak students when asked if the study curriculum accomplished the goal of training a professional with integrity (89% vs 66% vs 64%, *p* < 0.001), respectively. In a multivariate analysis graduating during the pandemic (OR 3.92; 95% CI, 2.58–5.94) and in the post-peak period (OR 4.24; 95% CI, 2.23–8.07) were independent factors for the appreciation that the study curriculum did not meet its objective.

**Conclusions:**

The pandemic has hindered medical education. Students’ appreciation of their instruction has deteriorated. Urgent interventions that halt the negative impact on training, ensure readiness for future problems and improve schooling worldwide are needed.

## Introduction

The emergence of a novel coronavirus and its associated disease named coronavirus disease 2019 (COVID-19) brought an unprecedented burden to the economy, education, and healthcare systems around the globe. The COVID-19 pandemic uncovered not only the deficiencies within the medical system, but also disrupted medical education and clinical care systems globally [1].

Most medical education programs in Ecuador are six-year programs. In the first two years, students learn about preclinical subjects, followed by three years of clinical education where they can practice in primary and secondary care facilities. The sixth year is an internship program where students rotate in the core areas of medicine such as internal medicine, surgery, gynecology and obstetrics, pediatrics, and the pre-rural service [2]. Before the COVID-19 pandemic, in-person learning was the only teaching modality in most medical schools in Ecuador.

To mitigate the impact of the ancestral severe acute respiratory syndrome 2 (SARS-CoV-2) strain, its associated variants, and sublineages, health authorities and governments implemented measures such as quarantine, isolation, and social distancing to halt transmission and decrease infections. Thus, social gathering and in-person learning, core aspects of the traditional education system, were interrupted [3]. For instance, the Association of American Medical Colleges decided to suspend clinical rotations [4]. While in many other countries like Ecuador, medical students were reassigned from in-person healthcare delivery to providing medical attention through telemedicine [5]. Thus, prompt and innovative educational measures had to be implemented, transitioning from bedside training to simulation-based instruction, asynchronous and synchronous distance education for guaranteeing continuity in medical education [6].

However, there are several limitations to online medical distance education aside from the inherent technological issues. Medical students develop limited communication skills and empathy due to the lack of opportunities to practice interviewing [6]. Also, online learning prevents medical students from acquiring the necessary hands-on training [6]. Moreover, several studies have documented the negative impact that the COVID-19 pandemic has had on medical students’ training [7–9]. Most students believed that online learning could not be used for clinical training, felt less prepared for postgraduate instruction, or considered their education inferior to that of prior generations [7–10].

To accurately evaluate the impact that the COVID-19 pandemic has had on medical students’ training in Ecuador, we surveyed senior medical students who completed their training before, during, and in the post-peak period of the COVID-19 pandemic. This study aimed to identify the attitudes of senior medical students regarding their education and compare them according to the three periods previously described.

## Methods

In this cross-sectional study, senior medical students from Pontificia Universidad Católica del Ecuador were asked to complete a web-based survey that was available between January 2018 and June 2022. Eligible responses were those of medical students that had completed the questionnaire.

A questionnaire designed to evaluate the medical curriculum from Pontificia Universidad Católica del Ecuador was used as a reference to develop the survey used in this study [11]. A multidisciplinary team of faculty members from that university selected specific questions from the original questionnaire to assess medical students' attitudes toward their medical career. The survey underwent a review process, during which the team evaluated its clarity and appropriateness and made any necessary amendments based on student feedback. The survey consisted of 19 questions that collected sociodemographic data, the modalities for obtaining the medical degree (i.e., final examination, thesis, essay), and the perception of medical students toward educational factors that aided in their professional traineeship. It also inquired if the university’s curriculum fulfilled the objective of “training a professional with integrity, academic excellence, research abilities, and social responsibility”. Besides, it explored if the university aided them to develop certain skills such as acting efficiently and within responsibility in society, becoming a leader and prone to solve primary care problems, diagnosing and promptly treating medical problems, promptly referring patients, assessing cost–benefit and risk–benefit care for patients, solving administrative and legal issues, using educational technology resources for work, and conducting research. Moreover, the questionnaire explored the attitudes that senior medical students had toward professors’ level of proficiency in medical subjects. And how satisfied they were with their academic formation. Responses were recorded on a three-point Likert scale (“slightly useful”, “moderately useful”, and “very useful”) or Yes/No questions.

Participants were classified into three groups considering their graduation year, the declaration of the COVID-19 pandemic by the World Health Organization (WHO) [12], and return to in-person classes in Ecuador [13]. The first group, from now on referred to as “pre-pandemic” were those medical students who graduated between 2018 and 2019. The second one, denominated “pandemic” were those who graduated between 2020 and 2021. The last group denominated “post-peak” where those who graduated in 2022 and returned to in-person learning.

As this study complies with the National Health Authority – Research Ethics Committee in Human Subjects regulations for observational studies, ethical approval for this study was not required [14].

As a secondary analysis of an existing dataset, we could not perform an a priori sample size calculation. However, we estimated the required sample size using the sample size calculator from the University of California San Francisco [15]. We used data from Manzar et al. and Al-Balas et al., who reported satisfaction rates among medical students of 42.8% and 26.77%, respectively [16, 17]. To detect a minimum difference in proportions of 16.03% of medical students satisfied with their education, with a statistical power of 80% and a type I error probability of 5%, a minimum of 300 participants were necessary.

### Data analysis

Descriptive statistics were used, including frequencies and percentages for categorical variables and means for quantitative variables. The Pearson’s chi-squared and Fisher’s exact tests were used to determine the association between medical students’ perception of their education and their COVID-19 group classification. Differences in mean values were analyzed using the one-way analysis of variance (ANOVA) test.

A binary logistic regression model was used to predict the risks of not feeling that the academic curriculum fulfilled the objective of training a professional with integrity based on independent variables such as sociodemographic characteristics, modalities for obtaining a medical degree, and the COVID-19 group classification.

The statistical analysis was carried out using IBM SPSS, version 25. All analyses were based on two-sided *p*-values, with *p* < 0.05 considered statistically significant.

## Results

A total of 804 responses were collected. After excluding duplicates and incomplete answers, 679 were considered valid and analyzed. The population included 281 (41%) men and 398 (59%) women with a median age of 25 (range 22–41). Most students considered themselves as mestizo (*n* = 666; 98%) and were single (*n* = 620; 91%). Senior medical students’ sociodemographic characteristics and their classification according to the declaration of the COVID-19 pandemic are detailed in Table 1. Up to 383, 241, and 55 senior medical students answered the survey before, during, and in the post-peak period of the COVID-19 pandemic, respectively. There were no significant differences in baseline sociodemographic characteristics between groups.

**Table 1:** Senior medical students' baseline sociodemographic characteristics

Variables		Overall population *n* = 679 (100%)	Pre-pandemic *n* = 383 (%)	Pandemic *n* = 241 (%)	Post-peak *n* = 55 (%)	*p*-value
**Sex**						0.561
	Male	281 (41)	161 (42)	101 (42)	19 (35)	
	Female	398 (59)	222 (58)	140 (58)	36 (65)	
**Age (median, range)**		25 (22–41)	25 (23–35)	25 (23–41)	24.5 (22–32)	0.182
**Race**						0.230
	White	9 (1)	5 (1)	1 (0)	3 (6)	
	Mestizo	666 (98)	375 (98)	239 (99)	52 (94)	
	Indigenous	2 (0)	1 (0)	1 (0)	0 (0)	
	Afro-descendant	1 (0)	1 (0)	0 (0)	0 (0)	
	Other	1 (0)	1 (0)	0 (0)	0 (0)	
**Marital status**						0.308
	Single	620 (91)	350 (91)	217 (90)	53 (96)	
	Married	39 (6)	24 (6)	13 (5)	2 (4)	
	Domestic partnership	16 (2)	6 (2)	10 (4)	0 (0)	
	Divorced	4 (1)	3 (1)	1 (1)	0 (0)	

Regarding the modalities for obtaining the medical degree, most students chose to take a final examination (*n* = 352; 52%), followed by writing a thesis (*n* = 296; 44%). Pre-pandemic students were less likely (49%) to take the final examination to obtain a medical degree compared to those from the post-peak period (69%) but more likely to write a thesis than the latter (48% vs 27%) (*p* = 0.003).

**Fig. 1:**
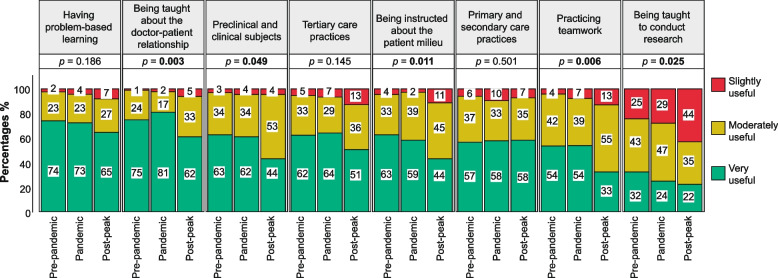
Factors that helped students during their professional traineeship

When senior medical students were asked about the factors that helped them during their professional traineeship, the top three features that were rated as “very useful” by most of the respondents were 1) being taught about the doctor-patient relationship (*n* = 518; 76%), 2) having problem-based learning (*n* = 496; 73%), and 3) tertiary care practices (*n* = 420; 62%). Also, there was a notable decrease in the percentage of students who considered certain factors as “very useful” during the post-peak period, compared to those from the pre-pandemic era. The subjects and factors with a significant decrease between the post-peak and pre-pandemic periods were being instructed about the patient milieu, being taught about the doctor-patient relationship, practicing teamwork, preclinical and clinical subjects, and being taught to conduct research. While having problem-based learning, primary, secondary, and tertiary care practices had a lower proportion of medical students considering “very useful” in the post-peak period than before the beginning of the pandemic. Figure 1 shows the students’ attitudes toward these factors and the differences according to the COVID-19 group classification.

**Fig. 2:**
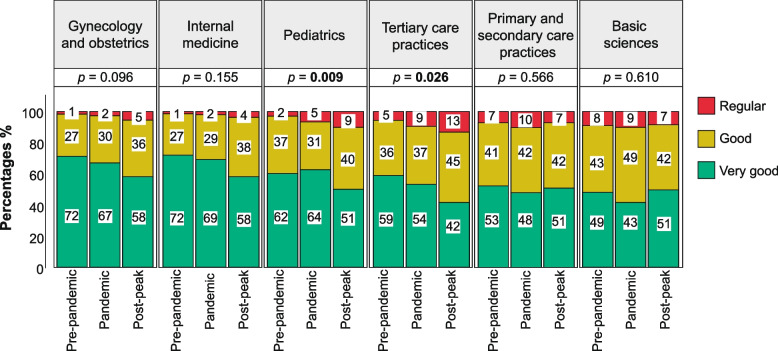
Appreciation of senior medical students toward professors’ proficiency

Figure 2 shows the appreciation of senior medical students toward professors’ level of proficiency in medical subjects and the differences according to the COVID-19 group classification. The top three subjects where professors were rated as “very good” by most of the students were internal medicine (*n* = 475; 70%), gynecology and obstetrics (*n* = 468; 69%), and pediatrics (*n* = 417; 61%). There was a significant decrease in the percentage of students who appreciated professors’ proficiency as “very good” in the post-peak period, compared to their pre-pandemic counterparts for pediatrics (51% vs 62%; *p* = 0.009) and tertiary care practices (42% vs 59%; *p* = 0.026). Additionally, there was a reduction in this same perception for other subjects such as gynecology and obstetrics, internal medicine, and primary and secondary care practices. Whereas basic sciences were the only theme that increased the percentage of students who appreciated professors’ proficiency as “very good” in the post-peak compared to the pre-pandemic period (51% vs 49%, *p* = 0.610).

**Fig. 3:**
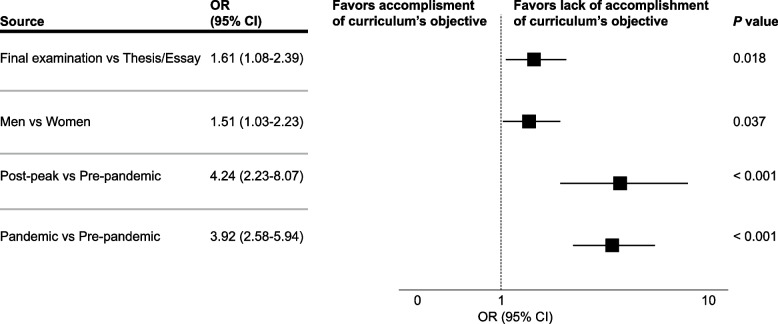
Multivariate logistic regression analysis to identify predictors for feeling that the study curriculum did not fulfill its objective

Moreover, students from the post-peak period and pandemic were less likely than pre-pandemic students to consider that the study curriculum accomplished the goal of training a professional with integrity, academic excellence, research abilities, and social responsibility (64% vs 66% vs 89%, *p* < 0.001), respectively. Furthermore, there was a significantly higher proportion of students from the pre-pandemic period that had high satisfaction with the medical career they cursed (75%) compared to those from the pandemic (60%) and post-pandemic (45%) period (*p* < 0.001). Univariate analyses showed that the variables associated with the perception that the study curriculum did not accomplish its primary goal included graduating during the pandemic (odds ratio [OR] 3.9; 95% CI, 2.58–5.89), graduating in the post-peak period (OR 4.4; 95% CI, 2.34–8.29), being male (OR 1.48; 95% CI, 1.02–2.14), and taking the final examination for obtaining the medical degree (OR 1.76; 95% CI, 1.21–2.57). Whereas the age and being single were not significant predictors (OR 0.99; 95% CI, 0.91–1.07) (OR 1.65; 95% CI, 0.76–3.58), respectively. Significant variables were included in a multivariate model and remained independent predictors for the perception that the study curriculum did not accomplish its primary goal (Fig. 3).

**Fig. 4:**
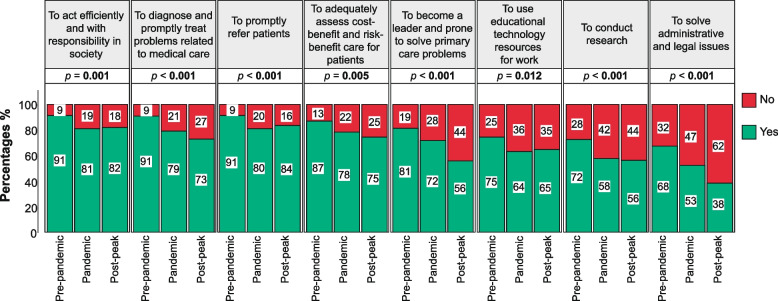
Appreciation of senior medical students toward the aid of the university to develop certain skills

Furthermore, there was a dramatic decrease in the percentage of post-peak students compared to those from the pre-pandemic period who thought that the university aided them to develop certain skills such as 1) acting efficiently and with responsibility in society (82% vs 91%; *p* = 0.001); 2) becoming a leader and prone to solve primary care problems (56% vs 81%; *p* < 0.001); 3) diagnosing and promptly treating problems related to medical care (73% vs 91%; *p* < 0.001); 4) promptly referring patients (84% vs 91%, *p* < 0.001); 5) adequately assessing cost–benefit and risk–benefit care for patients (75% vs 87%, *p* = 0.005); 6) solving administrative and legal issues (38% vs 68%, *p* < 0.001); 7) using educational technology resources for work (65% vs 75%, *p* = 0.012); and 8) conducting research (56% vs 72, *p* < 0.001). The percentages of medical students who believed that the university aided them to develop certain skills and the differences according to the COVID-19 group classification are shown in Fig. 4.

**Fig. 5:**
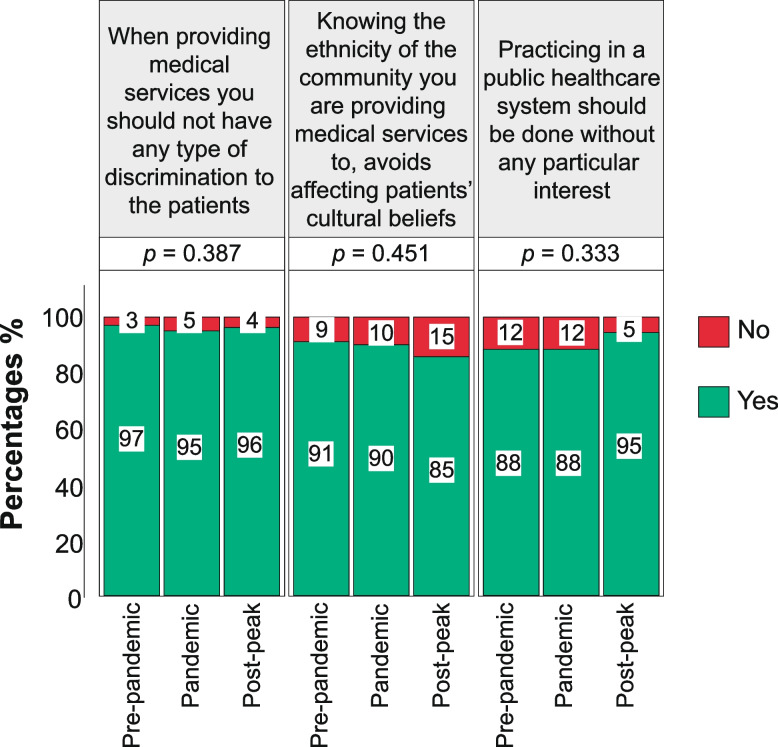
Core values and critical opinions of senior medical students

Nevertheless, as shown in Fig. 5, there were no significant differences between students graduating before the beginning of the pandemic, during the pandemic, and in the post-peak period when asked if they were aware that 1) when providing medical services they should not have any type of discrimination to the patients (97% vs 95% vs 96%, *p* = 0.387); 2) knowing the ethnicity of the community they were providing medical services to, avoids affecting patients’ cultural beliefs (91% vs 90% vs 85%, *p* = 0.451); 3) practicing in a public healthcare system should be done without any particular interest (88% vs 88% vs 95%, *p* = 0.333).

## Discussion

To the best of our knowledge, this is the first study to assess the attitudes of medical students toward their education before, during, and after the post-peak period of the COVID-19 pandemic. The results of this study add to the vast literature about the profound negative impact that the pandemic has had on medical education. And it raises concern regarding the lack of improvement in students’ education despite returning to pre-pandemic learning strategies.

The doctor-patient relationship has been the backbone of medicine. Learning about its importance is essential in the formation of a medical doctor. This sacred bond is a keystone of care due to the positive impact it has on medical decision-making and patient healing [18]. Moreover, another pillar in medical formation is problem-based learning. This learning modality has already proved to be superior to traditional methods for teaching medicine [19]. Additionally, tertiary care practices and hands-on training are a cornerstone in medical formation. As well known, medical students acquire essential clinical skills through observation, practice, and repetition under the guidance of mentors in clinical settings [7, 9, 20]. Despite these three factors being crucial to learning how to treat a patient, there was a decrease in the percentage of students who consider them helpful during their professional traineeship, particularly among those who have undergone training during the pandemic and post-peak period. Medical schools and authorities should take notice of these findings and foster the learning and implementation of these pillars in the current education models.

Although there is still an ongoing debate regarding the best learning modality (i.e., online vs in-person learning vs hybrid), it is well known that the lack of hands-on training has had a staggering detrimental impact on medical formation. While some studies have documented the repercussion that the pandemic has had on clinical training [6, 9, 21], it is important not to assume that only this group of students has been affected. As shown by this study, there was an overwhelming decrease in the percentage of students who perceived their preclinical and clinical training as “very useful” during the pandemic and post-peak period, compared to those from the pre-pandemic era. This could be attributed to several factors, which include the lack of interactive learning, limited patient exposure, increased academic burden, technological barriers, lack of motivation from students and professors, and insufficient online training for professors [1, 6, 22–28].

The astounding decrease in the percentage of students who appraise that the university curriculum fulfilled its primary objective in the pandemic and post-peak groups, compared to the pre-pandemic group, is disheartening and makes one think if the current appreciation will improve with time or remain unaltered. Data from this study show that students who graduated during the pandemic and post-peak period were 3.92 and 4.24 times more likely than their pre-pandemic counterparts to believe that the study curriculum did not fulfill its objective. However, it is paramount to remember that post-peak students spent a great part of their training during the pandemic, albeit graduating after the removal of public health and social measures. Noteworthy, students from the pandemic and post-peak period showed lower levels of preparedness compared to the pre-pandemic group in various areas such as diagnosing, treating, and promptly referring patients, assessing cost–benefit and risk–benefit care, solving administrative and legal issues, and conducting research. This is of utmost importance and should be noted by residency programs, as their upcoming residents could not have the appropriate skills and should receive training courses along with their usual instruction.

The preparedness of professors to swiftly transition to online learning is another crucial factor to explore. Recent investigations have shown that professors favor in-person over online lectures due to the improved interaction with students and feedback received [25, 26]. Additionally, the lack of training made it more challenging for them to prepare for virtual classes, causing them to spend more time and effort and resulting in feelings of inadequacy in providing the theoretical training they aimed to deliver, [26, 27] which could impact student learning and preparation. A study by Kim et al. found lower mean examination scores for students who took exams in 2020 compared to 2018 and 2019 [26]. While correlation does not necessarily imply causation, our findings support this reasoning, as students from the pandemic and post-peak period deemed that professors were less proficient in subjects such as gynecology and obstetrics, internal medicine, primary, secondary, and tertiary care practices compared to their pre-pandemic counterparts. Providing professors with the necessary tools and training for online teaching is urgently required.

Thinking that the pandemic has outgunned only medical schools in low- and middle-income countries due to the lack of technological resources (e.g., good internet quality and access to virtual online communication platforms) is ludicrous. Studies conducted in Libya, Mexico, the United Kingdom, the United States, and Jordan, upper-middle- and high-income countries according to the World Bank [29], suggest that even medical students from those countries consider that their education was disrupted by the pandemic and felt less prepared to take postgraduate examinations or practice their profession [7, 9, 10, 17, 21, 28]. However, it should be no surprise to anybody that low-income countries struggled far more than prosperous economies. Servin-Rojas et al. found these disparities evident within the same country, where private schools in Mexico were less likely than public schools to cancel their rotations due to the pandemic [9]. Likewise, in Nigeria, a survey involving medical students revealed that 75% of respondents stated that before the pandemic their institution did not use e-learning platforms, with most of the remaining percentage were from private schools. Even more alarming is that when the Government of Nigeria ordered the closure of educational institutions in March 2020, only 45% of schools could continue providing medical education [30]. Strategies that tackle these disparities both between and within countries are eagerly awaited.

Despite the drawbacks and challenges posed by the pandemic on medical education, it is pleasant and encouraging to see that core medical values such as non-discrimination toward any patient and practicing in the public healthcare system without particular interest are still deeply ingrained in medical students, regardless of the period when they graduated. Thus, highlighting that the commitment, intrinsic values, and qualities required to become a doctor have not been affected by this pandemic.

Since the beginning of the COVID-19 pandemic, global attention has focused on the number of infections, excessive death toll, and the development of vaccines and treatments. On September 14th, 2022, Dr. Tedros Adhanom Ghebreyesus, Director-General of the WHO, said: “The world has never been in a better position to end the pandemic. We are not there yet, but the end is in sight” [31]. Hence, as the light at the end of the tunnel finally comes into sight, it is time to focus on ending the COVID-19 pandemic and targeting problems that had been put aside. In short, only limited efforts have been taken to correctly assess the impact that the pandemic has had and will continue to have on medical education. As accurately stated by The *Lancet* Commission on lessons for the future from the COVID-19 pandemic: “Success also requires preparedness” [32], and we are in debt with education. Urgent efforts are needed to understand medical education difficulties, how to tackle them, and develop innovative solutions that can be replicated globally.

### Limitations

Among the limitations of this study, it should be considered its cross-sectional nature, reliance on self-reported data, a single-center experience which could restrict the generalizability of the results, the absence of a validated questionnaire which could limit the validity and reliability of the data gathered in this study, and the inability to measure educational outcomes (i.e., the number of applicants who entered residency programs) between groups. Moreover, the post-peak group had limited in-person training after the Ecuadorian government lifted remaining public health and social measures which allowed students to go back to in-person learning.

### Recommendations for future practice

Education authorities and regulators should focus on providing easy and free online learning resources to students, train educators, and provide the necessary resources to optimize online learning and foster hands-on training and mentorship to senior medical students in their clinical practices. Of note, residency programs should identify incoming trainees’ weak areas and provide the resources to improve them. Henceforth, attention should focus on designing a solid education system capable of effectively transitioning from in-person to online learning in case of the emergence of new viruses or immune evasive SARS-CoV-2 variants. More studies are needed to assess if medical education improves in the forthcoming years, and objectively assess the repercussions that the pandemic has had on students’ training.

## Conclusion

The negative impact that the COVID-19 pandemic has had on medical education and training is staggering. Students’ appreciation of their instruction has remarkably deteriorated during this pandemic and most likely will take long before it could reach pre-pandemic levels. The overarching repercussions that the pandemic has had on training are yet to be fully understood, most likely in the upcoming years. Urgent interventions are needed to improve schooling worldwide. Novel educational strategies should halt the pandemic’s negative impact on training, tackle shortfalls and failures, and ensure readiness for future problems.

## Data Availability

The datasets used and/or analyzed during the current study will be made available from the corresponding author upon request by qualified scientific and medical researchers for legitimate research purposes. Investigators are invited to submit study proposal requests detailing research questions and hypotheses to receive access to these data.

## Abbreviations:

ANOVA: One-way analysis of variance
COVID-19: Coronavirus disease 2019
CI: Confidence intervals
OR: Odds ratio
SARS-CoV-2: Severe acute respiratory syndrome coronavirus 2
WHO: World Health Organization
